# Cross-species analysis links cell-cell communication rewiring to NOTCH2 during serous endometrial carcinogenesis

**DOI:** 10.64898/2026.09.01.748540

**Published:** 2026-09-20

**Authors:** Matalin G Pirtz, Andrea Flesken-Nikitin, Coulter Q Ralston, Daryl J Phuong, Nating Wang, Tinyi Chu, Songru Chen, Yalin Zheng, Elisa Schmoeckel, Anna Yemelyanova, Ie-Ming Shih, Charles G Danko, Benjamin D Cosgrove, Alexander Yu Nikitin

## Abstract

Cell-cell interactions shape the fate of mutant cells during cancer initiation but how these interactions evolve during progression to pathologically recognizable lesions remain poorly understood. Here, we investigated cell-cell communication during serous endometrial carcinoma (SEC; also known as uterine serous carcinoma) development using a lineage-traceable mouse model and cross-species analyses of the mouse and human neoplastic endometrium. In mice, the early, pre-dysplastic stage was marked by a global decrease in inferred cell-cell interactions, followed by extensive communication network rewiring during neoplastic progression. Pathway-specific analysis revealed a similar pattern for NOTCH signaling, with NOTCH2 emerging as the dominant NOTCH receptor in Trp53/Rb1-mutant immature epithelial cells. Functionally, NOTCH2 promoted the outgrowth of more proliferative mutant organoids. Cross-species transcriptomic analysis identified conserved immature epithelial states in mouse and human neoplastic endometrial epithelium. In human tissues, NOTCH2 was overexpressed in serous endometrial intraepithelial carcinoma, a precursor of SEC, and in overt SEC. Furthermore, elevated NOTCH2 expression was associated with poor patient survival. These findings link cell-cell communication rewiring during experimental SEC development to conserved neoplastic epithelial states and identify NOTCH2 as an early marker and a potential target of disease interception.

## Full Text Availability

The license terms selected by the author(s) for this preprint version do not permit archiving in PMC. The full text is available from the preprint server.

